# Efficacy of weekly amrubicin for refractory or relapsed non-small cell lung cancer: A protocol of systematic review and meta-analysis

**DOI:** 10.1097/MD.0000000000020454

**Published:** 2020-06-19

**Authors:** Dong Dang, Chao Jiang, Ming-rui Xie

**Affiliations:** a Department of Oncology, Yulin Hospital of Traditional Chinese Medicine, Yulin; b The Third Department of Neurology, The Second Affiliated Hospital of Xi’an Medical University, Xi’an; c Department of Emergency, Longhua Hospital Shanghai University of Traditional Chinese Medicine, Shanghai; d Department of Oncology, Yulin No.2 Hospital, Yulin, China.

**Keywords:** amrubicin, efficacy, non-small cell lung cancer, safety

## Abstract

**Background:**

The aim of this study is to examine the efficacy of weekly amrubicin (WA) for treating refractory or relapsed non-small cell lung cancer (RRNSCLC).

**Methods:**

The literature search will be performed using the Cochrane Library, MEDLINE, EMBASE, CINAHL, PsycINFO, Scopus, Chinese Biomedical Literature Database, WANGFANG, VIP database, and China National Knowledge Infrastructure from inception onwards up to the March 1, 2020. No language limitation will be implemented. Randomized controlled trials that examined the efficacy and safety of WA for the treatment of RRNSCLC will be included. Literature selection, data extraction, and methodological quality assessment will be handled by 2 independent authors. We will invite a third author to disentangle any divergences between 2 authors. We will carry out statistical analysis using RevMan 5.3 software.

**Results:**

This study will summarize current evidence to assess the efficacy and safety of WA for the treatment of RRNSCLC.

**Conclusions:**

The findings of this study will provide helpful evidence for the clinician, and will promote further studies, as well as clarify the direction of research on WA for the management of RRNSCLC.

Study registration number: INPLASY202040168.

## 1 Introduction

Lung cancer is one of the most common malignant tumors, and is also the leading cause of cancer-related death globally.^[1–3]^ It is reported that there are about 2.1 million new patients and 1.8 million deaths of lung cancer around the world in 2018.^[4–6]^ Of those, about 85% of all lung cancer patients have non-small cell lung cancer (NSCLC).^[7–9]^ Although a variety of treatments are available for NSCLC, there are still some patients who develop to the refractory or relapsed non-small cell lung cancer (RRNSCLC).^[10–12]^

Weekly amrubicin (WA) is reported to treat RRNSCLC.^[13–22]^ However, all findings are based on the individual trial, and no systematic review has been reported to assess the WA for the treatment of RRNSCLC. Thus, this study will systematically evaluate the efficacy and safety of WA for RRNSCLC.

## 2 Methods

### 2.1 Study registry

We registered this study in the INPLASY202040168. We have prepared this study according to the statement of Preferred Reporting Items for Systematic Reviews and Meta-Analysis Protocols.^[23]^

### 2.2 Eligibility criteria for including studies

#### 2.2.1 Types of studies

In this study, we will only consider randomized controlled trials focusing on the efficacy and safety of WA for the treatment of RRNSCLC for inclusion. Any other types of studies, such as animal studies, case reports, case series, and review will all be excluded.

#### 2.2.2 Types of interventions

##### 2.2.2.1 Experimental group

All patients in the experimental group received WA for their treatment in this study.

##### 2.2.2.2 Control group

The participants in the control group could receive any therapies, except any types of WA.

#### 2.2.3 Types of patients

All adult patients (>18 years) who were diagnosed as having RRNSCLC regardless their sex, and educational and economic background will all be considered for inclusion.

#### 2.2.4 Types of outcome measurements

Primary outcomes are overall survival (defined as the time from randomization to death from any causes), and pathological complete response (defined as the complete disappearance of the invasive cancer in the lung and absence of tumor in the axillary lymph nodes).

Secondary outcomes include progression-free survival, recurrence-free survival, disease-free survival, quality of life, and any expected or unexpected adverse events.

### 2.3 Literature sources and search

We will perform literature searches using the following electronic bibliographic databases from their inception onwards to the March 1, 2020: Cochrane Library, MEDLINE, EMBASE, CINAHL, PsycINFO, Scopus, Chinese Biomedical Literature Database, WANGFANG, VIP database, and China National Knowledge Infrastructure. We will not establish any limitations to language and publication status. The search strategy sample with detailed information of Cochrane Library is presented in Table 1. In addition, similar search strategies will be adapted to the other electronic databases.

**Table 1:**
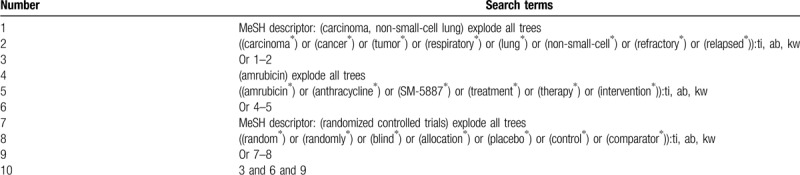
Search strategy sample of Cochrane Library.

At the same time, we will search grey literature sources, such as conference abstracts, clinical trial registries, and reference lists of previous reviews.

### 2.4 Study selection

All duplicated studies will be imported into Endnote X7 software and excluded before the screening. Two authors will independently scan all the records from title and abstract and all irrelevant literatures will be removed. Then, full manuscripts of all remaining studies will be further identified to check if they meet all inclusion criteria. We will note all excluded citations with specific reasons. If there are any different opinions between 2 authors, we will invite another author for consultation and final decision will be made after discussion. The process of study selection will be shown in a flow diagram.

#### 2.4.1 Data extraction

Two authors will independently extract the following associated information from each included trial: first author, time of publication, location, sample size, randomization methods, blinding, concealment, allocation, details of intervention and controls, number of sessions, duration of each session, duration of follow-up, outcome measurement tools, and any other relevant information. A third senior author will help to reconcile any divergences between 2 authors.

#### 2.4.2 Missing data dealing with

If we identify any unclear or missing data, we will contact original authors to obtain them. If we cannot get reply, we will only analyze available data and will discuss its potential affect as limitation.

#### 2.4.3 Quality assessment

Two authors will independently undertake study quality assessment using Cochrane risk of bias tool, which assesses potential biases in 7 domains. Each one is further determined as low, unclear, or high risk of bias. A third senior author will reconcile any different views between two authors.

#### 2.4.4 Subgroup analysis

We will preside over subgroup analysis to explore any potential heterogeneity and inconsistency based on the different characteristics of trial and patient, intervention and controls, and outcome measurement tools.

#### 2.4.5 Sensitivity analysis

We will consider running sensitivity analysis to identify the robustness and stability of merged results by excluding studies with high risk of bias.

#### 2.4.6 Reporting bias

If necessary, we will examine the reporting bias using funnel plot and Egger regression test when >10 trials are included.^[24,25]^

### 2.5 Data synthesis

We will undertake RevMan 5.3 software to analyze data and to perform meta-analysis if it is necessary. We will calculate all continuous data using mean difference or standardized mean difference and 95% confidence intervals. As for dichotomous data, we will exert it using risk ratio and 95% confidence intervals. All heterogeneity across included trials will be identified using *I*^*2*^ statistics. *I*^*2*^ ≤50% indicates low heterogeneity, and a fixed-effect model will be utilized for data pooling. However, *I*^*2*^ > 50% means high heterogeneity, and a random-effect model will be used for data synthesizing. Additionally, subgroup analysis will be operated to explore any possible reasons for the high heterogeneity. Whenever it is possible, we will conduct meta-analysis if at least 3 eligible criteria are fulfilled. Otherwise, meta-analysis will not be carried out if only 1 or 2 studies meet the inclusion criteria. Under such situation, the findings will be presented in a narrative summary. We will perform narrative synthesis if running meta-analysis is inappropriate due to the high heterogeneity. All narrative descriptions will be carried out based on the Guidance on the Conduct of Narrative Synthesis in Systematic Reviews.^[26]^

## 3 Discussion

This study will systematically analyze the current evidence for the efficacy and safety of WA for the treatment of RRNSCLC. Its strength is to examine a wider range of electronic databases to avoid missing any potential trials. In addition, the findings obtained in the present study may be beneficial in both clinical practice and health-related policy maker. Furthermore, it will also help to promote further studies and clarify the direction for the future research.

On the contrary, this study has several potential drawbacks. There may be a language bias, although there is not language limitation in this study. Moreover, the overall quality for some studies may be low, which may affect the findings of this study. Finally, some eligible trials may have small sample size, which may also impact this study.

## Author contributions

**Conceptualization:** Dong Dang, Chao Jiang, Ming-rui Xie.

**Data curation:** Dong Dang, Chao Jiang, Ming-rui Xie.

**Formal analysis:** Dong Dang, Ming-rui Xie.

**Investigation:** Ming-rui Xie.

**Methodology:** Dong Dang, Chao Jiang.

**Project administration:** Ming-rui Xie.

**Resources:** Dong Dang, Chao Jiang.

**Software:** Dong Dang, Chao Jiang.

**Supervision:** Ming-rui Xie.

**Validation:** Dong Dang, Chao Jiang, Ming-rui Xie.

**Visualization:** Dong Dang, Chao Jiang, Ming-rui Xie.

**Writing – original draft:** Dong Dang, Ming-rui Xie.

**Writing – review & editing:** Dong Dang, Chao Jiang, Ming-rui Xie.
